# The relationship between Cho/NAA and glioma metabolism: implementation for margin delineation of cerebral gliomas

**DOI:** 10.1007/s00701-012-1418-x

**Published:** 2012-06-23

**Authors:** Jun Guo, Chengjun Yao, Hong Chen, Dongxiao Zhuang, Weijun Tang, Guang Ren, Yin Wang, Jinsong Wu, Fengping Huang, Liangfu Zhou

**Affiliations:** 1 Shanghai Medical College, Fudan University, Shanghai, 200040 China; 2 Glioma Surgery Division, Department of Neurologic Surgery, Huashan Hospital, Shanghai Medical College, Fudan University, No.12 Central Wulumuqi Road, Jing’an District, Shanghai, 200040 China; 3 Department of Neuropathology, Fudan University, Shanghai, 200040 China; 4 Department of Radiology, Huashan Hospital, Shanghai, China

**Keywords:** Cho/NAA, Glioma boundary, ^1^H-MRSI, Tumour infiltration, Needle biopsy, Metabolism

## Abstract

**Background:**

The marginal delineation of gliomas cannot be defined by conventional imaging due to their infiltrative growth pattern. Here we investigate the relationship between changes in glioma metabolism by proton magnetic resonance spectroscopic imaging (^1^H-MRSI) and histopathological findings in order to determine an optimal threshold value of choline/*N*-acetyl-aspartate (Cho/NAA) that can be used to define the extent of glioma spread.

**Method:**

Eighteen patients with different grades of glioma were examined using ^1^H-MRSI. Needle biopsies were performed under the guidance of neuronavigation prior to craniotomy. Intraoperative magnetic resonance imaging (MRI) was performed to evaluate the accuracy of sampling. Haematoxylin and eosin, and immunohistochemical staining with IDH1, MIB-1, p53, CD34 and glial fibrillary acidic protein (GFAP) antibodies were performed on all samples. Logistic regression analysis was used to determine the relationship between Cho/NAA and MIB-1, p53, CD34, and the degree of tumour infiltration. The clinical threshold ratio distinguishing tumour tissue in high-grade (grades III and IV) glioma (HGG) and low-grade (grade II) glioma (LGG) was calculated.

**Results:**

In HGG, higher Cho/NAA ratios were associated with a greater probability of higher MIB-1 counts, stronger CD34 expression, and tumour infiltration. Ratio threshold values of 0.5, 1.0, 1.5 and 2.0 appeared to predict the specimens containing the tumour with respective probabilities of 0.38, 0.60, 0.79, 0.90 in HGG and 0.16, 0.39, 0.67, 0.87 in LGG.

**Conclusions:**

HGG and LGG exhibit different spectroscopic patterns. Using ^1^H-MRSI to guide the extent of resection has the potential to improve the clinical outcome of glioma surgery.

## Introduction

Delineating the boundaries of cerebral gliomas plays a vital role in glioma surgery because maximal resection of gliomas contributes greatly to prolonged survival, reduced rates of recurrence and morbidity [5, 29]. For the purpose of treatment planning, the extent of glioma is generally based on post-gadolinium MRI, together with T1- or T2-weighted magnetic resonance (MR) images [11]. The boundaries of tumour invasion are difficult to define because of the characteristic infiltrative growth pattern of gliomas. Contrast enhancement on T1-weighted images only illustrates the locations where the blood–brain barrier is compromised. Tumour cell infiltration could be detected over an area measuring from 6 to 14 mm from the outer area adjacent to the tumour edge as defined by the post-contrast MR imaging (MRI) [37]. Other studies, using biopsy findings to confirm histopathological data, reported that the extent of spread of glioma exceeded that defined by T2-weighted signal change [10, 31]. Thus, in general terms, routine anatomical imaging with MRI sequence techniques cannot be relied upon to indicate the true extent of spread of gliomas. Proton MR spectroscopy (^1^H-MRS) imaging (^1^H-MRSI) has been used in a number of studies to obtain biochemical information about local cellular metabolism. The technique, based on chemical shift imaging (CSI), facilitates characterisation of the tumour and surrounding normal brain tissue by determining the metabolic ratios of choline-containing compounds (Cho), *N*-acetyl-aspartate (NAA) and creatine (Cr) that are detected in the spectra [16, 33]. Compared with a normal brain, the signal of Cho is often elevated in the presence of tumorous tissue, which is thought to be due to increased membrane synthesis in rapidly dividing tumour cells [18, 26]. NAA, which is recognised as a putative internal neuronal marker, is decreased due to neuronal loss or dysfunction [7]. The Cr peak is the signal from both Cr and phosphocreatine and plays a role in the tissue energy metabolism [12]. Compared with single-voxel ^1^H-MRS, multi-voxel ^1^H-MRS is advanced in detecting the spatial distribution of metabolic changes in brain lesions because of its successive feature. It provides consecutive information about biochemical transformations in areas with low tumour infiltration and can be used to assist treatment planning [16, 33]. Better understanding of the relationships between ^1^H-MRS findings and glioma metabolism may enable physicians to distinguish normal tissue from infiltrated parenchyma in glioma.

Here we investigate the relationship between Cho/NAA ratio and MIB-1, p53, CD34 and tumour infiltration in order to evaluate the ability of Cho/NAA ratio to provide a unique parameter for glioma delineation.

## Materials and methods

### Patients

The study population included 18 patients (12 men and 6 women) with a mean age of 49.3 years (range, 18–69 years) who had newly diagnosed supratentorial gliomas. None of the patients had previously undergone surgery or received chemotherapy or radiotherapy. Details of the types and locations of tumours are summarised in Table 1.

**Table 1:** Type, grade, and location of the investigated gliomas in 18 eligible subjects

Subject no./sex/age(years)	Tumour type	WHO grade	Tumour location
1/M/59	Anaplastic astrocytoma	III	Right occipital
2/M/61	Glioblastoma multiforme	IV	Right temporo-occipital
3/F/54	Glioblastoma multiforme	IV	Left frontal
4/F/18	Astrocytoma	II	Left frontal
5/M/48	Glioblastoma multiforme	IV	Multipule occupation
6/M/46	Oligoastrocytoma	II	Right frontal
7/M/52	Anaplastic atrocytoma	III	Right frontotemporal
8/F/46	Oligoastrocytoma	II	Left frontal
9/M/56	Anaplastic astrocytoma	III	Right frontal
10/M/69	Astrocytoma	II	Right frontal
11/F/54	Oligoastrocytoma	II	Right frontal
12/F/41	Anaplastic astrocytoma	III	Right Parietal
13/M/53	Glioblastoma multiforme	IV	Left Insula
14/M/38	Oligoastrocytoma	II	Right frontal
15/M/40	Glioblastoma multiforme	IV	Left temporo-occipital
16/M/58	Anaplastic oligodendroglioma	III	Right frontal
17/M/41	Anaplastic astrocytoma	III	Left frontal
18/F/54	Astrocytoma	II	Right frontal

*WHO* World Health Organisation

The study was approved by the Huashan Committee on Human Research at the University of Fudan. Informed consent was obtained from all patients.

### MRI and spectroscopy

#### Conventional MRI

Each patient underwent an MRI and spectroscopy examination less than 24 h prior to surgery. The MRI studies were performed using an intraoperative MRI (MAGNETOM Verio 3.0 T, Siemens, Germany) integrated neurosurgical suite (IMIRIS, Winnipeg, Canada) equipped with an eight-channel head coil. T1- and T2-weighted images were acquired before ^1^H-MRS was performed.

The protocol for conventional MRI consisted of a sagittal T1-weighted fluid-attenuated inversion-recovery sequence (T1FLAIR, TR/TE/TI 2,000/9/860 ms), an axial T2-weighted turbo spin echo sequence (TSE, TR/TE 6,000-7,540/95-98 ms), axial T1-weighted fluid-attenuated inversion-recovery sequence (T1FLAIR, TR/TE/TI 2,000/9/860 ms), axial T2-weighted fluid-attenuated inversion-recovery (T2FLAIR, TR/TE/TI 8,500/94/2,440 ms), and axial T1-weighted contrast-enhanced gradient echo sequence (GRE, TR/TE 2,000/9 ms). A three-dimensional (3D) anatomic magnetisation prepared rapid acquisition gradient echo sequence (MPRAGE, TR/TE/TI 1,900/2.94/900 ms, FOV 250 mm × 250 mm, 1 mm isotropic, and 176 slices) or turbo spin echo sequence (TSE, TR/TE 3,200/332 ms, FOV 250 mm × 250 mm, slice thickness 2.0 mm, and 64 slices) was performed to obtain a neuron-navigation MRI data sets for the lesion either with or without contrast enhancement.

For registration to the frameless stereotactic system, six to eight adhesive skin fiducial marks were placed in a scattered pattern on the head surface. Before imaging, an 18- or 20-gauge intravenous catheter was inserted in the antecubital area as a contrast agent (Gadodiamide, GE Healthcare Ireland, 0.1 mmol/kg body weight) administration.

#### ^1^H-MRSI

The proton CSI raw data were achieved using the multivoxel point-resolved spectroscopy sequence (PRESS, TR/TE 1,700/135 ms, 15-mm section thickness, 16 × 16 phase-encoding steps, FOV 120 mm × 120 mm) after obtaining the contrast-enhancing images. Water suppression was achieved by using three chemical shift-selective pulses prior to the PRESS excitation. The position of the CSI slice was chosen to cross the largest diameter of the lesion on the T2-weighted images. The volume of interest (VOI) was positioned to include the enhancing lesion or abnormal signal region on the T2-weighted MRI, peritumoral region and normal contralateral brain, while avoiding contamination from scalp fat and skull lipid. The chemical shift artefact was minimised by four positioned regional saturation pulses. The resulting nominal spectroscopic voxels measured 7.5 mm × 7.5 mm × 15 mm.

The total ^1^H-MRS acquisition required approximately 25 min. At the end of the proton CSI data acquisition, the raw data file and scout images were exported to the post processing workstation (Syngo MultiModality Workplace, Siemens Healthcare, Siemens, Germany).

### ^1^H-MRS data analysis

**Fig. 1:**
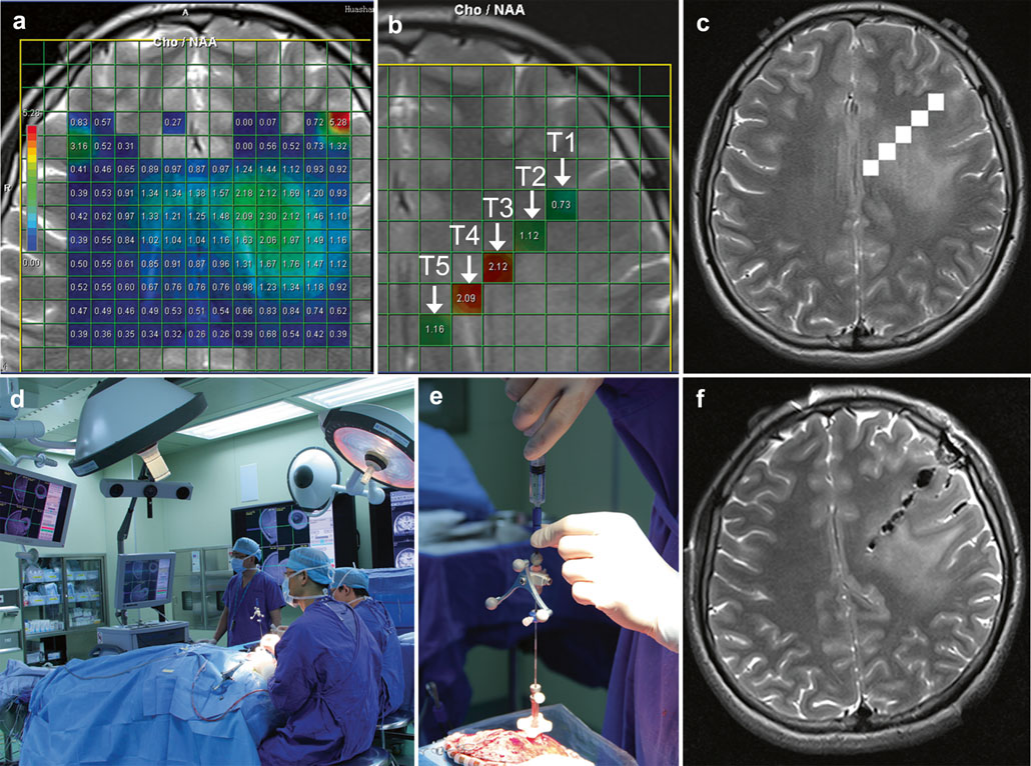
**a** T2-weighted MR image superimposed with coloured voxels from patient 4 with grade II astrocytoma. **b** Five voxels (T1, T2, T3, T4, T5) with IDs 197, 182, 167, 152, 137 corresponded to Cho/NAA ratio 0.73, 1.12, 2.12, 2.09, 1.16 respectively arranged from right to left were chosen by referring to the conventional MR images and local metabolic information together. **c** The five voxels were labelled in the neuronavigation data sets. **d** Positioning and calibration of navigation probe. **e** Skull-mounted trajectory guide guarantees the direction of biopsy needle. **f** The accurate sampling for needle biopsy was confirmed by intraoperative MR scanning

The raw spectral data were reconstructed using spectroscopy (Siemens Healthcare, Siemens, Germany). The spatial distribution of the metabolite of interest was generated by fitting curve to peak area. Peak parameters (height, width, area) for Cho and NAA were estimated on a voxel- by-voxel basis within each VOI and expressed as integral ratios. Cho/NAA values were displayed with a rainbow-type colour look-up table, whereby blue-green-yellow-red corresponded to values from zero to maximum. A peak information map on the scout image was displayed using an overlaid grid, which indicated the anatomical location from which the results had been derived (Fig. 1a). The overlaid grid consisted of 256 voxels (16 × 16) and each voxel was assigned an identification (ID), which started from the top left.

### Selection and label of biopsy targets

Each lesion was subdivided into three regions: tumour core (TC), immediate peritumoral region (IPR) and distant peritumoral region (DPR). TC was defined as the region with contrast enhancement on T1-weighted images, the hypointense region on T1-weighted images or hyperintense region on T2-weighted images in gliomas without contrast enhancement. The IPR was chosen as one voxel (7.5 mm on the transverse plane and 10.6 mm on the diagonal plane) distance perpendicular to the most outer margin of the TC. DPR was chosen as one to two voxels perpendicularly distant from the most outer margin of the TC. Meanwhile, three to seven targets in non-eloquent regions for tumour biopsy were preoperatively determined by referring to the conventional MR images and MR spectroscopic features of the lesion and the surgical trajectory was planned with the operator (J.S.W.). These biopsy targets were located at the TC, IPR and DPR. The location (ID) of each of the biopsy targets was recorded. In most situations a linear path was adopted so that the biopsy needle was inserted along the same track to reduce the extra brain injury.

The MRS raw data (rda file) and neuronavigation MR data sets were transferred to an MAC Pro (Apple) computer for analysis.

Biopsy_NAV software, developed at our laboratory (W.J.T.) for automatic labelling, was run in a Matlab (7.7.0 [R2008b]) environment. The IDs of the biopsy targets were entered followed successively by the rda and neuronavigation files (Fig. 1b). Neuronavigation data sets with labelled marks were generated automatically, and were subsequently viewed using medical imaging software OsiriX (v.3.7.1 32-bit) and sent to a Picture Archiving and Communication System (PACS; Fig. 1c).

### Tissue sampling

Biopsies were conducted within the dedicated intraoperative MRI integrated neurosurgical suite (IMRIS, Winnipeg, Canada intra-MRI Integrated Neurosurgical Suite). The neuronavigational MRI data sets with the labelled marks were transferred to the planning workstation of the surgical navigation system (StealthStation Treon, Medtronic, Minneapolis, MN, USA) and image fusion was performed, with other MRI sequences that had been obtained beforehand, using StealthMerge software (Medtronic, Minneapolis, MN, USA) (Fig. 1d).

To minimise the effects of potential brain shift, the biopsies were sampled prior to resection of the lesion under the guidance of neuronavigation using a Passive Biopsy Needle Kit (Medtronic, Minneapolis, MN, USA) which was tracked by the navigation system. A burr hole was made by referring to the trajectory made beforehand, and a skull-mounted trajectory guide was used to align and stabilise the biopsy needle before passing it through the brain to obtain samples (Fig. 1e). The operator targeted the centre of the voxel for biopsy specimen retrieval so that the samples would exactly match the ^1^H-MRSI. Each sampling location was recorded during the course of their extraction by using the screen save feature of the surgical navigation system and was renamed. Intraoperative MR scanning was subsequently performed to confirm the accuracy of the needle biopsy (Fig. 1f).

### Histopathological evaluation

Each biopsy sample was fixed in 10 % formalin and sent to the Neuropathology Department of Huashan Hospital. The samples were embedded in paraffin and cut into sections for histopathological assessment.

Haematoxylin and eosin (H & E)-stained sections of all tumours were reviewed under a light microscope (OLYMPUS, BX50) by two blinded neuropathologists (H.C. and Y.W.) and categorised according to the fourth edition of the World Health Organisation Classification of Tumours of the Central Nervous System (2007).

**Fig. 2:**
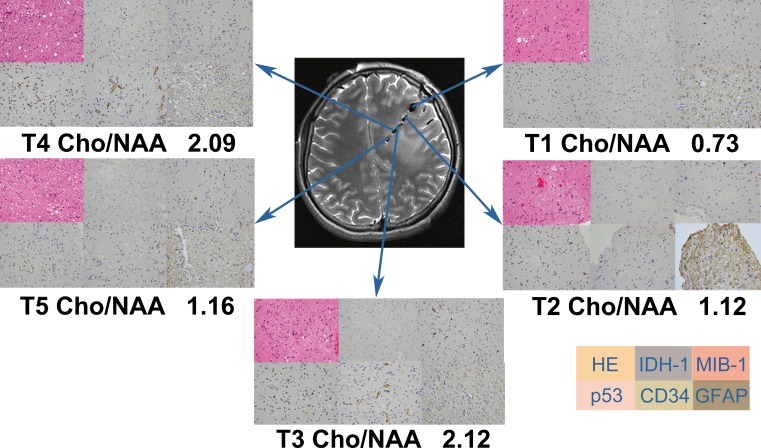
H & E (×400) and immunohistochemical staining with IDH-1, MIB-1, p53, CD34 and GFAP antibodies (×400, respectively) were performed respectively in each biopsy sample. The maximal Cho/NAA ratio (2.12) corresponded to the maximal value of cell density, MIB-1 and p53. The minimal Cho/NAA ratio (0.73) corresponded to the minimal value of cell density, MIB-1, and p53. All specimen were strong immunopositive for GFAP and moderate immunopositive for CD34, but immunonegative for IDH-1

Immunohistochemistry was performed using the EnVision method with diaminobenzidine (DAB) as a chromogen. The primary antibodies were: IDH1 R132H (Dianova, Hamburg, Germany, clone H09, monoclonal, 1:200), MIB-1 (Dako, clone MIB-1, monoclonal, 1:200), p53 (Dako, clone DO-7, monoclonal, 1:200), CD34 (Dako, monoclonal, 1:200) and GFAP (Dako, clone 6 F2, monoclonal, 1:200) (Fig. 2).

IDH1 expression was categorised as being negative or positive based on the negatively or positively stained cell cytoplasm or cell membrane. The MIB-1 labelling index (LI) representing tumour cell proliferation was calculated as the percentage of positively stained nuclei and was classified as: strong (MIB-1 ≥ 10 %), moderate (5 ≤ MIB-1 <10 %), mild (1 ≤ MIB-1 <5 %), or negative (MIB-1 <1 %).

Evaluation of the p53, CD34 and GFAP expression was divided into four groups according to the percentage of positively stained tumour cells: strong (> 50 %, +++), moderate (10–50 %, ++), mild (< 10 %, +), and negative (−).

**Fig. 3:**
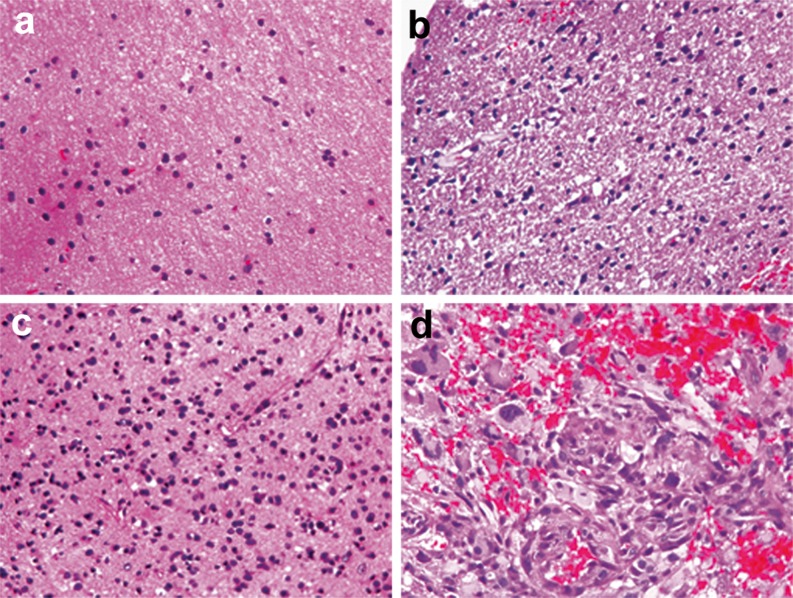
Criteria for tumour infiltration. **a** Without tumour infiltration (normal brain or gliosis) (×400). **b** Mild tumour infiltration (low cell density, mild nuclear atypia) (×400). **c** Moderate tumour infiltration (moderate cell density, obvious nuclear atypia, few mitoses) (×400). **d** Heavy tumour infiltration (high cell density, obvious nuclear atypia, abundant mitoses) (×400)

Observation of cell morphology (magnification ×200) was performed at five random fields of concentrated tumour cells. Information was collected on cell density, nuclear abnormalities and mitosis. Tumour infiltration into each biopsy specimen (Fig. 3) was categorised as: (1) no tumour infiltration (normal brain or gliosis, MIB-1 <1 %); (2) mild tumour infiltration (low cell density, mild nuclear abnormalities, 1 ≤ MIB-1 <5 %); (3) moderate tumour infiltration (moderate cell density, obvious nuclear abnormalities, few mitoses, 5 ≤ MIB-1 <10 %); (4) marked tumour infiltration (high cell density, obvious nuclear abnormalities, abundant mitoses, MIB-1 ≥10 %). IDH1 was used to ascertain whether the specimen was infiltrated with glioma cells or not when debate persisted on the basis of histopathological criteria.

### Statistical analysis

All statistical analyses were undertaken using SAS version 9.2 software. Kruskal-Wallis test was performed to compare the Cho/NAA ratios of biopsy samples from different regions. The relationship between dependent variables (MIB-1, p53, CD34, tumour infiltration) and independent variable (Cho/NAA) was calculated using Logistic Regression (SAS PROC LOGISTIC). Specimens were categorised into tumour or non-tumour tissue to further define HGG and LGG specimens with tumour infiltration. The probability of HGG and LGG specimens containing a tumour cells is calculated using logistic regression functions when Cho/NAA is 0.5, 1.0 1.5, 2.0.

*P* values ≤ 0.05 were considered statistically significant for all tests.

## Results

A total of 91 biopsy specimen loci were labelled and documented on the 3D neuronavigation MRI data sets (Fig. 1). Image-guided needle biopsies yielded 82 samples and 686 observations. A mean of four to five tissue samples (range, three to seven) and 37 observations per patient were obtained (Fig. 2). The histological classification identified seven cases of LGG and 11 cases of HGG (including six grade III gliomas and five grade IV gliomas).

The number and percentage of biopsy samples at different biopsy locations and histological classification of the individual biopsy specimens in HGG and LGG are summarised in Tables 2 and 3, respectively. Fifty-one biopsy samples collected from 11 patients with HGG comprised 22 HGG biopsies (17 grade III gliomas and five grade IV gliomas), 19 LGG biopsies and 10 samples without neoplastic tissue. Each case contained at least two biopsy samples with different histological grades. The Cho/NAA ratios from these samples at TC, IPR, DPR were 4.1, 1.04, 0.73 respectively. The difference between locations was statistically significant (*P* < 0.0001, Kruskal-Wallis test).

**Table 2:** Classification and location of the specimen from HGG

WHO grade of specimens	TC (%) (*n* = 32)	IPR (%) (*n* = 14)	DPR (%) (*n* = 5)
Non-tumour	0 (0. %)	6 (43 %)	4 (80 %)
LGG	13 (41 %)	5 (36 %)	1 (20 %)
HGG	19 (59 %)	3 (21 %)	0 (0 %)

**Table 3:** Classification and location of the specimen from LGG

WHO grade of specimens	TC (%) (*n* = 18)	IPR (%) (*n* = 8)	DPR (%) (*n* = 5)
Non-tumour	1 (6 %)	6 (75 %)	4 (80 %)
LGG	17 (94 %)	2 (25 %)	1 (20 %)

Thirty–one biopsy samples collected from seven patients with LGG were composed of 20 LGG samples and 11 samples without neoplastic tissue. One biopsy sample that did not contain any tumour cells came from the TC of a grade II glioma. The Cho/NAA ratio from these samples at TC, IPR, DPR were 2.375, 1.13, 0.900 respectively. The difference of Cho/NAA between these locations was also statistically significant(*P* < 0.0001, Kruskal-Wallis test).

The number and percentage of HGG and LGG biopsy samples with negative, mildly positive, moderately positive and strongly positive staining with MIB-1, p53, CD34, GFAP and IDH-1 antibodies are shown in Table 4. One of the specimens from LGG was categorised as gliosis based on IDH1 immunonegative when uncertainty remained regarding histopathology.

**Table 4:** Number and percentage of biopsy samples with immunohistochemical staining with MIB-1, p53, CD34, GFAP and IDH-1 antibodies

Dependent variable	HGG (*n* = 51)				LGG (*n* = 31)			
	Negative (%)	Mild (%)	Moderate (%)	Strong (%)	Negative (%)	Mild (%)	Moderate (%)	Strong (%)
MIB-1	11 (21 %)	22 (43 %)	9 (18 %)	9 (18 %)	16 (52 %)	14 (45 %)	1 (3 %)	0 (0. %)
p53	28 (55 %)	22 (43 %)	1 (2 %)	0 (0. %)	23 (73 %)	3 (10 %)	2 (7 %)	3 (10 %)
CD34	7 (14 %)	36 (70 %)	4 (8 %)	4 (8 %)	1 (4 %)	30 (96 %)	0 (0. %)	0 (0. %)
GFAP	0 (0. %)	2 (4 %)	19 (38 %)	30 (58 %)	0 (0. %)	2 (6 %)	11 (36 %)	18 (58 %)
IDH-1	35 (68 %)	16 (32 %)	0 (0. %)	0 (0. %)	13 (42 %)	18 (58 %)	0 (0. %)	0 (0. %)

Logistic regression analysis identified Cho/NAA as an independent variable and MIB-1, p53, CD34 and tumour infiltration as response variables. The relationship between variables MIB-1, p53, CD34, tumour infiltration and Cho/NAA was analysed in HGG and LGG samples separately. The relationships between MIB-1, p53, CD34, tumour infiltration and Cho/NAA in HGG are summarised in Table 5. No correlation was found between Cho/NAA and MIB-1, p53, C34, tumour infiltration in LGG.

**Table 5:** Statistics on the relationship between Cho/NAA and CD34, MIB-1, p53 and degree of tumour infiltration in HGG

Dependent variable	Parameter	Slope	*P* value	OR value
MIB-1	Cho/NAA	0.341	0.001	1.407
p53	Cho/NAA	0.037	0.332	1.038
CD34	Cho/NAA	0.179	0.016	1.196
Tumour infiltration	Cho/NAA	0.316	0.041	1.372

Statistically significant at *P* < 0.05

Additional logistic regression analysis undertaken to categorise specimens into tumour or non-tumour tissue, and thereby further define specimens with tumour infiltration, resulted in statistically significant *P* values for the variable “tumour” of 0.0379 in HGG and 0.0315 in LGG samples. The logistic regression functions regarding variables “Cho/NAA” and variable “tumour” in HGG and LGG are as follows.

$$ Ln\left( {\frac{{P\left( {\text{tumour}} \right)}}{{1 - P\left( {\text{tumour}} \right)}}} \right) = - 1.372 + 1.791 \times Cho/NAA\quad \quad Ln\left( {\frac{{P\left( {\text{tumour }} \right)}}{{1 - \left( {\text{tumour}} \right)}}} \right) = - 2.807 + 2.347 \times Cho/NAA $$

A Cho/NAA ratio threshold value of 0.5, 1.0 1.5 and 2.0 respectively was found to predict specimens containing a tumour cells with a probability of 0.38, 0.60, 0.79, 0.90 in HGG samples and 0.16, 0.39, 0.67, 0.87 in LGG samples.

## Discussion

MRS findings have been shown be closely related to histological features of glioma cells and can be used in tumour differentiation, grading, follow-up and radiotherapy planning [4, 14, 21, 27]. MRS is also a valuable tool for identifying early changes in glioma metabolism and the extent of glioma infiltration [19, 22, 31, 32]. The Cho/NAA ratio has been found to provide a sensitive method for detecting differences in tumour growth, and provides more reliable results than the Cho to *N*-acetyl aspartate index (CNI) or the Cho/Cr ratio [16].

It has been reported that MIB-1 may be the best index for predicting the potential of tumour proliferation, tumour grade and outcome [24]. CD34 has been shown to be closely related to angiogenesis, which is a key determinant in the progression of glioma [9, 34, 35]. MIB-1 and CD34 staining in our study showed strongly positive results in HGG of 18 % and 8 % respectively. There was much less staining in LGG tissues as shown in Table 4. These findings suggest that HGG are more aggressive and more highly vascularised than LGG. The MRS results were also different between HGG and LGG. Logistic regression analysis indicated that higher Cho/NAA ratios were associated with a high MIB-1 labelling index (*P* = 0.001) and stronger CD34 expression (*P* = 0.0155) in HGG, no correlation was found in LGG. expression.

Other workers have demonstrated a linear correlation between Cho and MIB-1 in gliomas that showed a homogeneous pattern with MRI scanning [13, 30]. Likewise, Matsumura et al. [15]. found a positive correlation between Cho and MIB-1 in benign glioma and inverse correlation in malignant gliomas. In our study we investigated the relationship between Cho/NAA and MIB-1, p53, CD34, tumour infiltration. Multi-voxel ^1^H-MRSI was used to avoid the limitations related to single-voxel ^1^H-MRSI and was able to detect the consecutive metabolism change of gliomas. It has also been proposed that the invasive and aggressive nature of malignant astrocytomas may be related to p53 abnormalities [20]. However, in our study no statistically significant association was found between p53 and Cho/NAA.

Many studies have focused on pursuing cut-off value of different metabolite ratios and have used these ratios in an attempt to contour gliomas. McKnight and collaborators conducted a study with ^1^H-MRSI to identify a CNI that predicted tumour tissue and ruled out normal tissues [16]. They confirmed these findings using stereotactic brain biopsies, and found that active tumours could be differentiated from normal, edematous, gliotic, or necrotic tissue with 90 % sensitivity and 86 % specificity by use of a CNI threshold of 2.5. Rock et al. [25] found that a Cho/normal creatine ratio of more than 1.79 or a Lip-Lac/normal creatine ratio of less than 0.75 was able to predict whether a spectroscopic voxel contained tumour or necrotic tissue. However, Ganslandt et al. [10] failed to find a common range of Cho/NAA ratios that were predictive for a given degree of tumour infiltration.

In our study, the metabolite ratio of Cho/NAA was found to predict whether or not a spectroscopic voxel contained tumour cells. We showed that specimens from patients with HGG contained tumour cells with a probability of 0.60 when the Cho/NAA ratio was 1.0 and with a probability of 0.67 in patients with LGG using a Cho/NAA ratio of 1.5. Different threshold values should, therefore, be adopted to delineate tumour margins in HGG and LGG. Our Cho/NAA ratio threshold value for distinguishing tumour from non-tumour in HGG was comparable to that reported by Widhalm et al. [36], who defined spectra as pathological when Cho/NAA was more than 1.0. The same workers described specific colour-coded visualisations of distinct intratumoral CSI maxima.

Both glioma grade and the location of glioma need to be considered when determining the glioma margin. More extensive resection may be required when the tumour is located in a non-eloquent region and in these cases the boundaries of the glioma resection should be based on a lower Cho/NAA ratio. Caution should be taken when the tumour is located in an eloquent region. For these cases, the resection margins should be based on a higher Cho/NAA ratio, in accordance with the functional MRI or intraoperative neurophysiological monitoring, to avoid the potential neurological deficits. A balanced approach aimed at increasing the extent of resection and decreasing morbidities, will lead to high quality survival.

To date, no unified criteria exist for assessing tumour infiltration. Stadlbauer et al. [33] defined glioma infiltration in terms of relative and absolute tumour cell numbers, with minimal infiltration being seen when this value was defined a relative tumour cell number <15 %. Croteau et al. [6] classified the degree of tumour infiltration into six levels based on tumour cellularity. Our own criteria for tumour infiltration were based on cell morphology in terms of cell density, nuclear abnormalities and mitosis. In additional to the traditional criteria for tumour infiltration, we introduced IDH1 into our classification criteria for infiltration to help distinguish LGG from gliosis at the glioma border zone, which is often difficult using traditional histopathological criteria. Compared with other established glioma markers (including GFAP, p53 and WT1) IDH1 has been shown to have high specificity and sensitivity in differentiating reactive gliosis from neoplastic cells and even detected a single infiltrating tumour cell at the infiltrating edge of the gliomas [1–3]. We therefore believe that the degree of tumour infiltration was evaluated accurately in our study.

**Fig. 4:**
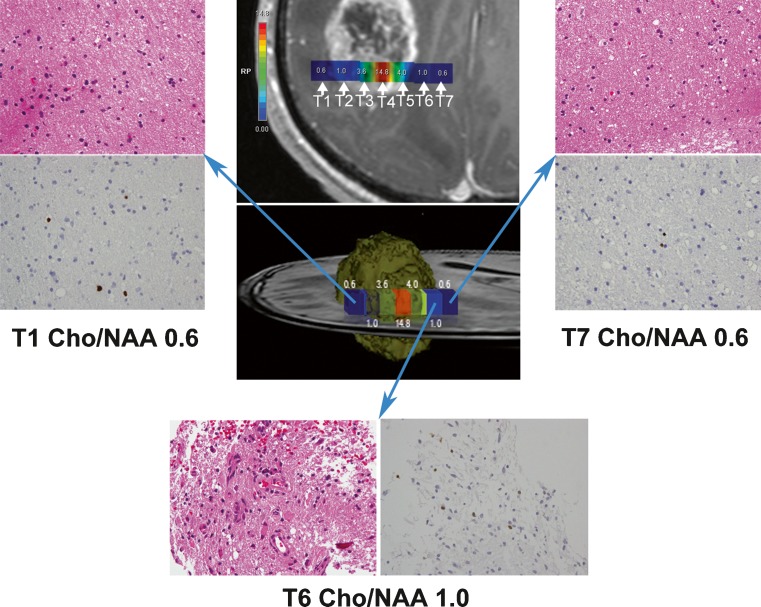
Contrast-enhanced T1-weighted image superimposed with coloured voxels (*upper middle*) from patient 5 with glioblastoma multiforme. Seven voxels (T1, T2, T3, T4, T5, T6, T7) with IDs 43, 59, 75, 91, 107, 123, 139 arranged from left to right. A 3-D ideograph of the case (*centre*) rendered using Photoshop. H & E stained sections (×400) and MIB-1 (×400) showed the infiltration degree of T1, T6 and T7. Both T1 (*upper left*) and T6 (*bottom*) were located in IPR but had different Cho/NAA ratios. Histopathology confirmed that specimens from T1 and T6 were infiltrated differently. T1 and T7 (*upper right*) were located in IPR and DPR respectively but they shared the same Cho/NAA ratio and histopathology confirmed that specimens from T1 and T7 were infiltrated similarly

A feature of our study was that the biopsy targets were consecutive, being located in the TC and tumour border (IPR and/or DPR). The use of consecutive biopsy targets is in accordance with the biological infiltrative behaviour of glioma. We showed that the Cho/NAA ratio was correlated with the location of the biopsy. The Cho/NAA ratios at the TC, IPR, DPR were respectively 4.1, 1.04, 0.73 in HGG and 2.375, 1.13, 0.900 in LGG. The differences in Cho/NAA ratios at different locations in HGG and LGG were statistically significant. The probability of IPR, DPR being infiltrated by glioma was respectively 57 % and 20 % with HGG and 25 % and 20 % with LGG. All of HGGs consisted of at least two different grade compositions. Thirteen biopsies from the TC in HGG patients were classified as grade II glioma, one biopsy from the TC of a LGG patient was classified as gliosis indicating that the glioma was profoundly heterogeneous. These findings were in agreement with McKnight et al.’s study [17]. Cell infiltration into the non-uniform boundaries of gliomas can therefore, be identified by the Cho/NAA ratio. We found that, although voxel T1 and voxel T7 were respectively located in IPR and DPR, they shared the same Cho/NAA ratio (Fig. 4), indicating that both regions were infiltrated to a similar extent. Pathological examination of the two specimens also demonstrated a similar degree of tumour infiltration. By contrast, voxel T1 and voxel T6 were both located in IPR, but displayed different Cho/NAA ratios. Histopathological validation showed that the specimens from these two voxels had different cellularity and MIB-1 expression and were infiltrated to different degrees. These results are in agreement with previous findings that define the extent of tumour cell infiltration using non-uniform margins rather than uniform margins [23]. Tumour cells are more prone to invade the brain tissue medial to the glioma. It has been proposed that the differences in tumour infiltration may be associated with different fibre orientations. Gliomas preferentially infiltrate along fibres but not perpendicular to the direction that the fibres run [28]. It is also possible that the degree of infiltration is related to the abundance of local neovessels. This might explain why glioma resection along the same distance perpendicular to the outer tumour margin can achieve gross total resection in one orientation and only partial resection in another, ultimately resulting in recurrence.

In our study we were able to accurately match histological specimens with voxels. It is usually difficult to label the ^1^H-MRSI information on navigational images due to the different formats of MRI data sets. However, the NAV_biopsy software developed at our laboratory enabled us to successfully label biopsy targets in the navigation sequence without the need for recording the coordinates of each voxel preoperatively. In other studies [8, 23], biopsy locations were tracked back to the exact voxel position in the MRI data sets by the frameless stereotactic software, which was not as precise as this study. Furthermore, in our study the specimens were first obtained through needle biopsy regardless of the type of craniotomy that would subsequently be performed. So brain shift was avoided. And skull-mounted trajectory guaranteed a more accurate biopsy than could be achieved freehand (Fig. 1).

Limitations of our study should, however, be noted. The ^1^H-MRS technique used to provide cellular metabolic information is influenced by the cell growth cycle. Cells in the growth stage can be detected by ^1^H-MRS but cells in quiescent stage cannot be detected due to their slow metabolism, which means that ^1^H-MRSI is not sensitive to metabolically silent areas. The voxel volumes in our study were large (7.5 × 7.5 × 15 mm) in comparison to the volumes of the specimen (1.0 × 1.0 × 3.0 mm). Smaller voxels would have allowed the ^1^H-MRS to reflect the specimen exactly. However, smaller voxels require a long acquisition time which is unbearable for some patients. Using higher field strengths may solve this problem in future studies.

## Conclusions

The preliminary results of our study demonstrated that Cho/NAA is closely related to MIB-1, CD34 and tumour infiltration in HGG. Compared with conventional MRI, ^1^H-MRSI can better reflect glioma metabolism and delineate the glioma boundaries. HGG and LGG exhibited different spectroscopic patterns and had different threshold values that can predict the probability of a specimen containing a tumour. Using ^1^H-MRSI to guide the extent of resection has the potential to significantly improve the rate of gross total resection and subsequently prolong the overall survival time.

## References

[1] Capper D, Sahm F, Hartmann C, Meyermann R, von Deimling A, Schittenhelm J (2010). Application of mutant IDH1 antibody to differentiate diffuse glioma from nonneoplastic central nervous system lesions and therapy-induced changes. Am J Surg Pathol.

[2] Capper D, Weissert S, Balss J, Habel A, Meyer J, Jager D, Ackermann U, Tessmer C, Korshunov A, Zentgraf H, Hartmann C, von Deimling A (2010). Characterization of R132H mutation-specific IDH1 antibody binding in brain tumors. Brain Pathol.

[3] Capper D, Zentgraf H, Balss J, Hartmann C, von Deimling A (2009). Monoclonal antibody specific for IDH1 R132H mutation. Acta Neuropathol.

[4] Chen J, Huang SL, Li T, Chen XL (2006). In vivo research in astrocytoma cell proliferation with 1H-magnetic resonance spectroscopy: correlation with histopathology and immunohistochemistry. Neuroradiology.

[5] Claus EB, Horlacher A, Hsu L, Schwartz RB, Dello-Iacono D, Talos F, Jolesz FA, Black PM (2005). Survival rates in patients with low-grade glioma after intraoperative magnetic resonance image guidance. Cancer.

[6] Croteau D, Scarpace L, Hearshen D, Gutierrez J, Fisher JL, Rock JP, Mikkelsen T (2001). Correlation between magnetic resonance spectroscopy imaging and image-guided biopsies: semiquantitative and qualitative histopathological analyses of patients with untreated glioma. Neurosurgery.

[7] De Stefano N, Matthews PM, Arnold DL (1995). Reversible decreases in N-acetylaspartate after acute brain injury. Magn Reson Med.

[8] Dowling C, Bollen AW, Noworolski SM, McDermott MW, Barbaro NM, Day MR, Henry RG, Chang SM, Dillon WP, Nelson SJ, Vigneron DB (2001). Preoperative proton MR spectroscopic imaging of brain tumors: correlation with histopathologic analysis of resection specimens. AJNR Am J Neuroradiol.

[9] Farin A, Suzuki SO, Weiker M, Goldman JE, Bruce JN, Canoll P (2006). Transplanted glioma cells migrate and proliferate on host brain vasculature: a dynamic analysis. Glia.

[10] Ganslandt O, Stadlbauer A, Fahlbusch R, Kamada K, Buslei R, Blumcke I, Moser E, Nimsky C (2005). Proton magnetic resonance spectroscopic imaging integrated into image-guided surgery: correlation to standard magnetic resonance imaging and tumor cell density. Neurosurgery.

[11] Kelly PJ, Daumas-Duport C, Kispert DB, Kall BA, Scheithauer BW, Illig JJ (1987). Imaging-based stereotaxic serial biopsies in untreated intracranial glial neoplasms. J Neurosurg.

[12] Kemp GJ (2000). Non-invasive methods for studying brain energy metabolism: what they show and what it means. Dev Neurosci.

[13] Kimura T, Sako K, Gotoh T, Tanaka K, Tanaka T (2001). In vivo single-voxel proton MR spectroscopy in brain lesions with ring-like enhancement. NMR Biomed.

[14] Liu X, Germin BI, Zhong J, Ekholm S (2010). N-Acetyl peak in MR spectra of intracranial metastatic mucinous adenocarcinomas. Magn Reson Imaging.

[15] Matsumura A, Isobe T, Anno I, Takano S, Kawamura H (2005). Correlation between choline and MIB-1 index in human gliomas. A quantitative in proton MR spectroscopy study. J Clin Neurosci.

[16] McKnight TR, Lamborn KR, Love TD, Berger MS, Chang S, Dillon WP, Bollen A, Nelson SJ (2007). Correlation of magnetic resonance spectroscopic and growth characteristics within Grades II and III gliomas. J Neurosurg.

[17] McKnight TR, von Dem BM, Vigneron DB, Lu Y, Berger MS, McDermott MW, Dillon WP, Graves EE, Pirzkall A, Nelson SJ (2002). Histopathological validation of a three-dimensional magnetic resonance spectroscopy index as a predictor of tumor presence. J Neurosurg.

[18] Miller BL (1991). A review of chemical issues in 1 H NMR spectroscopy: N-acetyl-L-aspartate, creatine and choline. NMR Biomed.

[19] Mirbahai L, Wilson M, Shaw CS, McConville C, Malcomson RD, Griffin JL, Kauppinen RA, Peet AC (2011). 1 H magnetic resonance spectroscopy metabolites as biomarkers for cell cycle arrest and cell death in rat glioma cells. Int J Biochem Cell Biol.

[20] Momota H, Narita Y, Matsushita Y, Miyakita Y, Shibui S (2010). p53 abnormality and tumor invasion in patients with malignant astrocytoma. Brain Tumor Pathol.

[21] Narayana A, Chang J, Thakur S, Huang W, Karimi S, Hou B, Kowalski A, Perera G, Holodny A, Gutin PH (2007). Use of MR spectroscopy and functional imaging in the treatment planning of gliomas. Br J Radiol.

[22] Park I, Bok R, Ozawa T, Phillips JJ, James CD, Vigneron DB, Ronen SM, Nelson SJ (2011). Detection of early response to temozolomide treatment in brain tumors using hyperpolarized 13 C MR metabolic imaging. J Magn Reson Imaging.

[23] Pirzkall A, Li X, Oh J, Chang S, Berger MS, Larson DA, Verhey LJ, Dillon WP, Nelson SJ (2004). 3D MRSI for resected high-grade gliomas before RT: tumor extent according to metabolic activity in relation to MRI. Int J Radiat Oncol Biol Phys.

[24] Quinones-Hinojosa A, Sanai N, Smith JS, McDermott MW (2005). Techniques to assess the proliferative potential of brain tumors. J Neurooncol.

[25] Rock JP, Hearshen D, Scarpace L, Croteau D, Gutierrez J, Fisher JL, Rosenblum ML, Mikkelsen T (2002). Correlations between magnetic resonance spectroscopy and image-guided histopathology, with special attention to radiation necrosis. Neurosurgery.

[26] Sabatier J, Gilard V, Malet-Martino M, Ranjeva JP, Terral C, Breil S, Delisle MB, Manelfe C, Tremoulet M, Berry I (1999). Characterization of choline compounds with in vitro 1 H magnetic resonance spectroscopy for the discrimination of primary brain tumors. Invest Radiol.

[27] Sankar T, Caramanos Z, Assina R, Villemure JG, Leblanc R, Langleben A, Arnold DL, Preul MC (2008). Prospective serial proton MR spectroscopic assessment of response to tamoxifen for recurrent malignant glioma. J Neurooncol.

[28] Schluter M, Stieltjes B, Hahn HK, Rexilius J, Konrad-verse O, Peitgen HO (2005). Detection of tumour infiltration in axonal fibre bundles using diffusion tensor imaging. Int J Med Robot.

[29] Shaw EG, Wisoff JH (2003). Prospective clinical trials of intracranial low-grade glioma in adults and children. Neuro Oncol.

[30] Shimizu H, Kumabe T, Shirane R, Yoshimoto T (2000). Correlation between choline level measured by proton MR spectroscopy and Ki-67 labeling index in gliomas. AJNR Am J Neuroradiol.

[31] Stadlbauer A, Buchfelder M, Doelken MT, Hammen T, Ganslandt O (2011). Magnetic resonance spectroscopic imaging for visualization of the infiltration zone of glioma. Cen Eur Neurosurg.

[32] Stadlbauer A, Moser E, Gruber S, Buslei R, Nimsky C, Fahlbusch R, Ganslandt O (2004). Improved delineation of brain tumors: an automated method for segmentation based on pathologic changes of 1 H-MRSI metabolites in gliomas. Neuroimage.

[33] Stadlbauer A, Nimsky C, Buslei R, Pinker K, Gruber S, Hammen T, Buchfelder M, Ganslandt O (2007). Proton magnetic resonance spectroscopic imaging in the border zone of gliomas: correlation of metabolic and histological changes at low tumor infiltration—initial results. Invest Radiol.

[34] Udani V, Santarelli J, Yung Y, Cheshier S, Andrews A, Kasad Z, Tse V (2005). Differential expression of angiopoietin-1 and angiopoietin-2 may enhance recruitment of bone-marrow-derived endothelial precursor cells into brain tumors. Neurol Res.

[35] Wang S, Fang J, Zhang T, Wang B, Chen J, Li X, Zhang S, Zhang W (2011) Magnetic resonance imaging targeting of intracranial glioma xenografts by Resovist-labeled endothelial progenitor cells. J Neurooncol 105:67-75 10.1007/s11060-011-0569-6 21523487

[36] Widhalm G, Krssak M, Minchev G, Wohrer A, Traub-Weidinger T, Czech T, Asenbaum S, Marosi C, Knosp E, Hainfellner JA, Prayer D, Wolfsberger S (2011). Value of 1H-magnetic resonance spectroscopy chemical shift imaging for detection of anaplastic foci in diffusely infiltrating gliomas with non-significant contrast-enhancement. J Neurol Neurosurg Psychiatry.

[37] Yamahara T, Numa Y, Oishi T, Kawaguchi T, Seno T, Asai A, Kawamoto K (2010). Morphological and flow cytometric analysis of cell infiltration in glioblastoma: a comparison of autopsy brain and neuroimaging. Brain Tumor Pathol.

